# Invasive Hemodynamic Assessment and Procedural Success of Transcatheter Tricuspid Valve Repair—Important Factors for Right Ventricular Remodeling and Outcome

**DOI:** 10.3389/fcvm.2022.891468

**Published:** 2022-06-02

**Authors:** Varius Dannenberg, Matthias Koschutnik, Carolina Donà, Christian Nitsche, Katharina Mascherbauer, Gregor Heitzinger, Kseniya Halavina, Andreas A. Kammerlander, Georg Spinka, Max-Paul Winter, Martin Andreas, Markus Mach, Matthias Schneider, Anna Bartunek, Philipp E. Bartko, Christian Hengstenberg, Julia Mascherbauer, Georg Goliasch

**Affiliations:** ^1^Division of Cardiology, Department of Internal Medicine II, Medical University of Vienna, Vienna, Austria; ^2^Department of Cardiac Surgery, Medical University of Vienna, Vienna, Austria; ^3^Department of Internal Medicine and Cardiology, Charité – Universitätsmedizin Berlin (Campus Virchow-Klinikum), Berlin, Germany; ^4^Department of Cardiology, German Heart Center Berlin, Berlin, Germany; ^5^Department of Anesthesia and Intensive Care, Medical University of Vienna, Vienna, Austria; ^6^Department of Internal Medicine 3, University Hospital St. Pölten, Karl Landsteiner University of Health Sciences, Krems, Austria

**Keywords:** transcatheter repair, pulmonary hypertension, right ventricular remodeling, patient selection, tricuspid regurgitation

## Abstract

**Introduction:**

Severe tricuspid regurgitation (TR) is a common condition promoting right heart failure and is associated with a poor long-term prognosis. Transcatheter tricuspid valve repair (TTVR) emerged as a low-risk alternative to surgical repair techniques. However, patient selection remains controversial, particularly regarding the benefits of TTVR in patients with pulmonary hypertension (PH).

**Aim:**

We aimed to investigate the impact of preprocedural invasive hemodynamic assessment and procedural success on right ventricular (RV) remodeling and outcome.

**Methods:**

All patients undergoing TTVR with a TR reduction of ≥1 grade without precapillary or combined PH [mean pulmonary artery pressure (mPAP) ≥25 mmHg, mean pulmonary artery Wedge pressure ≤ 15 mmHg, pulmonary vascular resistance ≥3 Wood units] were assigned to the responder group. All patients with a TR reduction of ≥1 grade and precapillary or combined PH were classified as non-responders. Patients with a TR reduction ≥2 grade were directly classified as responders, and patients without TR reduction were directly assigned as non-responders.

**Results:**

A total of 107 patients were enrolled, 75 were classified as responders and 32 as non-responders. We observed evidence of significant RV reverse remodeling in responders with a decrease in RV diameters (−2.9 mm, *p* = 0.001) at a mean follow-up of 229 days (±219 SD) after TTVR. RV function improved in responders [fractional area change (FAC) + 5.7%, *p* < 0.001, RV free wall strain +3.9%, *p* = 0.006], but interestingly further deteriorated in non-responders (FAC −4.5%, *p* = 0.003, RV free wall strain −3.9%, *p* = 0.007). Non-responders had more persistent symptoms than responders (NYHA ≥3, 72% vs. 11% at follow-up). Subsequently, non-response was associated with a poor long-term prognosis in terms of death, heart failure (HF) hospitalization, and re-intervention after 2 years (freedom of death, HF hospitalization, and reintervention at 2 years: 16% vs. 78%, log-rank: *p* < 0.001).

**Conclusion:**

Hemodynamic assessment before TTVR and procedural success are significant factors for patient prognosis. The hemodynamic profiling prior to intervention is an essential component in patient selection for TTVR. The window for edge-to-edge TTVR might be limited, but timely intervention is an important factor for a better outcome and successful right ventricular reverse remodeling.

## Graphical Abstract

Influence of TR reduction and pulmonary hypertension on RV remodeling and outcome after transcatheter tricuspid edge-to-edge repair. TR, tricuspid regurgitation; PH, pulmonary hypertension; RV, right ventricle; TAPSE, tricuspid annulus plane systolic excursion; FAC, fractional area change; TTVR, transcatheter tricuspid valve repair; HF, heart failure. 
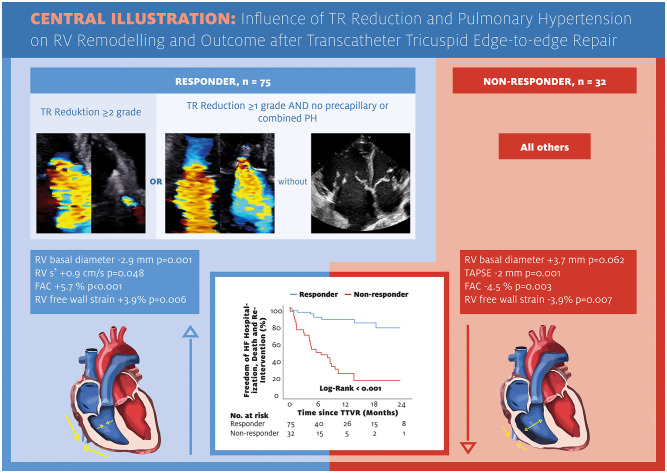

## Introduction

Tricuspid regurgitation (TR) is a common condition in the general population. Around 2% are affected by at least moderate TR, compared to 23% in patients with heart failure (HF) (1, 2). Severe TR is associated with increased hospitalization rates due to right heart failure and death (3–6). TR is mostly secondary and can develop in combination with left-sided valvular heart disease and as an isolated valvular lesion (7). Besides medical therapy, surgery has long been the only treatment, but isolated tricuspid valve surgery is associated with increased perioperative mortality (8, 9). Several devices for transcatheter tricuspid valve repair (TTVR) have been recently introduced to clinical practice, but transcatheter edge-to-edge repair is currently the most commonly used method (10). Several prospective observational studies have shown that TTVR can improve symptoms, right ventricular function, and outcome but might be unfavorable in patients with pulmonary hypertension (PH) (11–13). Based on these results, the European Society of Cardiology (ESC) implemented a 2b recommendation for TTVR in the 2021 guidelines for the management of patients with valvular heart disease (14). However, the ACC/AHA guidelines published in 2020 did not include a recommendation for TTVR due to missing evidence (15). Therefore, further studies and randomized controlled trials (RCT) are needed to firmly establish TTVR in the treatment of TR. The study focuses on i) the outcome of TTVR patients separated into different PH groups, ii) the effects of TR reduction and PH on outcome and RV remodeling after TTVR, iii) the (pre)procedural conditions for improved outcome and RV remodeling after TTVR.

## Materials and Methods

### Study Design and Study Population

**Figure 1:**
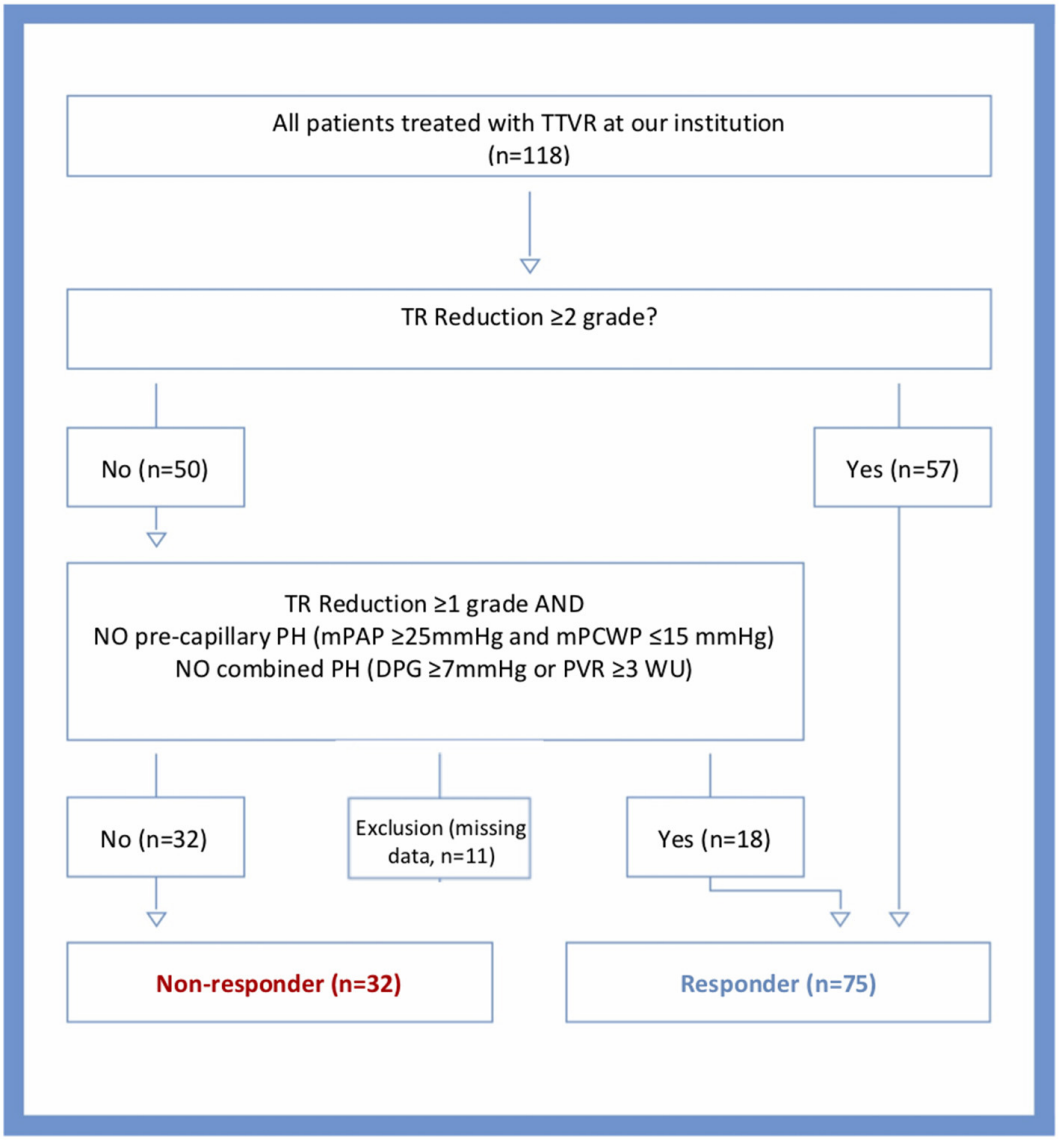
Algorithm for the allocation of patients to responders or non-responders. TTVR, transcatheter tricuspid valve repair; TR, tricuspid regurgitation; PH, pulmonary hypertension; mPCWP, mean pulmonary capillary Wedge pressure; DPG, diastolic pulmonary gradient; PVR, pulmonary vascular resistance; WU, Wood units.

We included all patients treated with edge-to-edge TTVR between September 2018 and December 2021 at the Medical University of Vienna. Patients were separately analyzed according to their PH group and were enrolled and classified as either responders or non-responders according to an algorithm illustrated in Figure 1. All patients undergoing TTVR with a TR reduction of ≥1 grade without precapillary or combined PH (mean pulmonary artery pressure (mPAP) ≥25 mmHg, mean pulmonary artery Wedge pressure ≤ 15 mmHg, pulmonary vascular resistance ≥3 Wood units) were assigned to the responder group. All patients with a TR reduction of ≥1 grade and precapillary or combined PH were classified as non-responders. Patients with a TR reduction ≥2 grade were directly classified as responders, and patients without TR reduction were directly assigned as non-responders. Baseline characteristics were recorded before the procedure. The multidisciplinary Heart Team of our center individually discussed and assigned all patients to TTVR based on current guidelines and recommendations. The study protocol was approved by the Ethics Committee of the Medical University of Vienna, and all patients consented to participate.

### Echocardiographic Assessment

A comprehensive echocardiographic assessment, including transesophageal echocardiography (TEE), was performed according to the American Society of Echocardiography guidelines (16, 17). Physicians and sonographers examined all patients using commercially available equipment (Vivid 7, E9, E95, GE Healthcare; and EPIQ 7, Philips Medical Systems), and board-certified physicians interpreted echocardiograms. Cardiac chamber sizes were evaluated according to the American Society of Echocardiography guideline recommendation (16). A comprehensive assessment of the tricuspid valve and TR was performed with an integrated, multiparametric approach, including the tricuspid valve morphology, vena contracta (VC), effective regurgitation orifice area (EROA), and regurgitant volume (RegVol) using the proximal isovelocity surface area (PISA) method (18). We applied a grading scale ranging from 1 to 5 to define TR severity: grade 1 indicates “mild”, 2 “moderate”, 3 “severe”, 4 “massive”, and 5 “torrential,” as recently proposed (19). Right ventricular systolic function was assessed using tricuspid annular plane systolic excursion (TAPSE), tissue Doppler velocity of the lateral tricuspid annulus (RV s'), fractional area change (FAC), and RV freewall strain and strainrate (20, 21). Systolic pulmonary artery pressure (sPAP_echo_) was calculated by adding the peak tricuspid regurgitation systolic gradient to the estimated central venous pressure (16). All analyses were performed using GE EchoPac software version 203 (GE Vingmed, Horten, Norway).

### Invasive Hemodynamic Assessment

Invasive hemodynamic assessment was performed routinely in study participants before TTVR. Hemodynamic measurements were performed using a 7F Swan-Ganz catheter (Edwards Lifesciences GmbH, Austria) *via* femoral access. Pressures were documented as the average of eight measurements over eight consecutive heart cycles using CathCorLX (Siemens AG, Berlin and Munich, Germany). In addition to pulmonary artery Wedge pressure (PAWP), the systolic (sPAP), diastolic (dPAP), and mean (mPAP) PA pressures were documented. Cardiac output (CO) was measured by Fick's method or thermodilution. If both were available, Fick's method was preferred. Furthermore, the transpulmonary gradient (TPG) and diastolic pulmonary vascular pressure gradient (DPG) were calculated according to current guidelines (22). TPG was computed by subtracting PAWP from mPAP; DPG was calculated as the difference between dPAP and PAWP during a pull-back; pulmonary vascular resistance (PVR) was calculated by dividing TPG by CO. Precapillary PH was defined as mPAP ≥25mmHg and mPCWP ≤ 15 mmHg and combined pre-/postcapillary PH was defined as DPG ≥7mmHg or PVR ≥3 WU (Figure 1) (22). Moreover, coronary angiography was performed in all patients to detect possible coronary artery disease.

### Procedural Characteristics

TTVR was performed using the Tri-/MitraClip (Abbott Laboratories, North Chicago, Illinois, size XT and XTW) or PASCAL system (Edwards Lifesciences, Irvine, California, size Ace). Both systems were inserted *via* a steerable guide with a delivery catheter through a right femoral vein access site. Precise valve anatomy and pathophysiology were assessed by transesophageal and transgastric echocardiographic windows using TEE according to recently published literature (23). The devices were positioned in the right atrium in front of the tricuspid valve. Steering of the guide and delivery catheter, rotation of the device arms, loading and grasping of the leaflets, device closure, and release were performed under fluoroscopic and echocardiographic guidance, as recently described (24).

The treating physician determined treatment strategy, device selection, and the number of implants based on the anatomic and clinical conditions of the individual patient.

### Outcome Analyses

Patients were followed up prospectively in a specialized outpatient clinic after TTVR at 3 months, 6 months, and annually. We defined the primary endpoint as all-cause mortality during a follow-up period of 2 years. In addition, we defined heart failure (HF) hospitalization as a secondary study endpoint and a composite endpoint, including death, HF hospitalization, and reintervention. Endpoints were collected *via* the Austrian death registry, telephone calls to patients or relatives, and electronic medical records. All patients gave written informed consent, and the study was approved by the Ethics Committee of the Medical University of Vienna.

### Statistical Analysis

Continuous baseline characteristics are presented for all patients and separately for the responder and non-responder groups as mean (SD) and compared with a 2-sided Student's *t*-test or Wilcoxon rank-sum test. Categorical variables were described as frequencies and compared with chi-square or Fisher's exact test. We compared follow-up data with baseline data for responders and non-responders, applying a paired Student's t-test or Wilcoxon rank-sum test. For different PH groups, we compared RV functional parameters at baseline and follow-up. Using described endpoints, Kaplan-Meier curves were plotted for all PH groups, responders, and non-responders. The log-rank test was applied to estimate the differences between survival curves. A two-sided *p*-value < 0.05 was considered statistically significant. Furthermore, univariate and multivariate logistic regression were performed using invasive hemodynamic data and patients with one or more and two or more grade TR reduction after TTVR. All analyses were performed using SPSS 27 (IBM SPSS, USA).

## Results

### Clinical Characteristics

A total of 118 patients were treated with TTVR at our institution between September 2018 and December 2021. One hundred and seven patients were included in the study, 75 in the responder group and 32 in the non-responder group. Eleven patients were excluded due to 1 grade TR reduction without invasive hemodynamic measurements. 35 patients had no PH, 2 had precapillary, 32 had postcapillary, and 19 had combined PH. Baseline data are displayed for all patients in Table 1 and for responders and non-responders in Table 2. The mean age of responders was 76 years, and 67% were female. In the non-responder group, the mean age was 77 years, and 59% were female. Concomitant transcatheter mitral valve repair (TMVR) was performed in 35% of the responders and in 47% of the non-responders. A significant difference in baseline characteristics between groups was in the presence of previous percutaneous coronary intervention (PCI, responders: 16% vs. non-responders: 36%, *p* = 0.022) and TRI-SCORE risk evaluation (responders: 14% vs. non-responders: 27%, *p* = 0.003) (25).

**Table 1:** Baseline Characteristics for all patients.

**Clinical characteristics**	***n =* 107**
Age, yrs	76 (9)
Female	69 (65)
NYHA ≤ 2	16 (15)
Leg edema	71 (66)
Coronary artery disease	44 (41)
Previous myocardial infarction	12 (11)
Previous PCI	24 (22)
Previous CABG	22 (21)
Previous valve surgery	22 (21)
Atrial fibrillation	96 (90)
CIED	33 (31)
Chronic lung disease	26 (24)
Cerebral vascular disease	12 (11)
Peripheral arterial disease	6 (6)
Hypertension	95 (89)
Diabetes	30 (28)
Dyslipidemia	55 (51)
eGFR, mL/min	45 (18)
NT-proBNP, ng/L	3,770 (4,428)
Bilirubin, mg/dL	0.88 (4.9)
EuroSCORE II, %	8.5 (6.8)
TRI-SCORE, %	18 (16)
**Pulmonary hypertension class**	
No PH	35 (40)
Precapillary PH	2 (2)
Postcapillary PH	32 (36)
Combined PH	19 (18)
**Procedural data**	
Concomitant TMVR	41 (38)
Baseline TR Vena contracta, mm	16 (5)
Baseline TR EROA, cm^2^	0.80 (0.54)
Baseline TR RegVol, mL	60 (26)
Residual TR Vena contracta, mm	8.5 (5.7)
Residual TR EROA, cm^2^	0.34 (0.34)
Residual TR RegVol, mL	25 (21)
TV inflow gradient, mmHg	1.3 (0.7)
**Echocardiography**	
RV basal diameter, mm	49.6 (8.8)
TV annulus, mm	43.1 (7.7)
TAPSE, mm	17.4 (5.5)
RV s', cm/s	10.2 (2.6)
FAC, %	40.2 (9.3)
RV enddiastolic area, cm^2^	26 (8.2)
RV endsystolic area, cm^2^	15.7 (6.0)
RA volume, ml	136 (21)
sPAP, mmHg	45 (14)
LVEF Simpson, %	52 (13)
RV free wall strain, %	20.9 (6.5)
RV free wall strain rate, 1/s	1.2 (0.4)
**Invasive hemodynamic measurements**	
sPAP, mmHg	43.7 (13.6)
dPAP, mmHg	17 (6.7)
mPAP, mmHg	27.3 (8.7)
mPCWP, mmHg	18.4 (6.9)
vRA, mmHg	16.4 (8.6)
mRA, mmHg	12.1 (6.4)
PVR, WU	2.7 (1.8)
DPG, mmHg	−1.5 (4.6)
TPG, mmHg	8.9 (5.2)

*Values are numbers (%) or mean (standard deviation)*.

*NYHA, New York Heart Association; PCI, percutanous coronary intervention; CABG, coronary artery bypass graft; CIED, cardiac implantable eletronic device; eGFR, estimated glomerular filtration rate; NT-proBNP, N-terminales pro-B-type natriuretic peptide; EuroSCORE, European Sytem for Cardiac Operative Risk Evaluation; PH, pulmonary hypertension; TMVR, transcatheter mitral valve repair; TR, tricuspid regurgitation; EROA, effective regurgitant orifice area; RegVol, regurgitant volume; TV, tricuspid valve; TAPSE, tricuspid annulus plane systolic excursion; RV, right ventricle; FAC, fractional area change; RA, right atrium; sPAP, systolic pulmonary artery pressure; LVEF, left ventricular ejection fraction; dPAP, diastolic pulmonary artery pressure; mPAP, mean pulmonary artery pressure; mPCWP, mean pulmonary capillary Wedge pressure; vRA, v-wave pressure right atrium; mRA, mean pressure right atrium; PVR, pulmonary vascular resistance; WU, Wood units; DPG diastolic pulmonary pressure gradient; TPG, transpulmonary pressure gradient*.

**Table 2:** Baseline characteristics by groups.

**Clinical characteristics**	**Responder**	**Non-responder**	***p***
	***n =* 75**	***n =* 32**	
Age, yrs	76 (10)	77 (7)	0.919
Female	50 (67)	19 (59)	0.512
NYHA ≤ 2	13 (17)	3 (9)	0.293
Leg edema	48 (64)	23 (72)	0.432
Coronary artery disease	29 (39)	15 (47)	0.521
Previous myocardial infarction	8 (11)	4 (13)	0.749
Previous PCI	12 (16)	12 (36)	**0.022**
Previous CABG	15 (20)	7 (22)	0.800
Previous valve surgery	13 (17)	9 (28)	0.295
Atrial fibrillation	67 (89)	29 (91)	1.000
CIED	24 (32)	9 (28)	0.820
Chronic lung disease	17 (23)	9 (28)	0.624
Cerebral vascular disease	7 (9)	5 (16)	0.338
Peripheral arterial disease	5 (7)	1 (3)	0.666
Hypertension	66 (88)	29 (91)	1.000
Diabetes	17 (23)	13 (41)	0.065
Dyslipidemia	37 (49)	18 (56)	0.534
eGFR, mL/min	47 (19)	41 (16)	0.180
NT-proBNP, ng/L	3,785 (4,362)	4,083 (4,896)	0.796
Bilirubin, mg/dL	0.85 (0.5)	0.96 (0.48)	0.291
EuroSCORE II, %	7.8 (6.8)	10 (6.8)	0.137
TRI-SCORE, %	14 (12)	27 (20)	**0.003**
Pulmonary hypertension class			0.133
No PH	27 (44)	8 (31)	
Precapillary PH	0 (0)	2 (8)	
Postcapillary PH	22 (36)	10 (39)	
Combined PH	13 (21)	6 (23)	
**Procedural data**			
Concomitant TMVR	26 (35)	15 (47)	0.280
Baseline TR Vena contracta, mm	16 (5)	17 (5)	0.516
Baseline TR EROA, cm^2^	0.77 (0.49)	0.85 (0.63)	0.769
Baseline TR RegVol, mL	60 (26)	60 (27)	0.992
Residual TR Vena contracta, mm	6 (3)	15 (5)	**<0.001**
Residual TR EROA, cm^2^	0.18 (0.14)	0.68 (0.37)	**<0.001**
Residual TR RegVol, mL	15 (11)	47 (22)	**<0.001**
TV inflow gradient, mmHg	1.2 (0.6)	1.4 (0.9)	0.354
**Echocardiography**			
RV basal diameter, mm	49 (8.3)	51.1 (10)	0.215
TV annulus, mm	42.3 (7.2)	44.9 (8.6)	0.114
TAPSE, mm	17.5 (5.5)	17 (5.7)	0.661
RV s', cm/s	10.6 (2.7)	9.3 (2.3)	**0.036**
FAC, %	40.7 (9.1)	39 (10)	0.406
RV enddiastolic area, cm^2^	25 (7.4)	28.9 (9.5)	**0.036**
RV endsystolic area, cm^2^	14.8 (5.1)	17.9 (7.4)	**0.035**
RA volume, ml	122 (59)	171 (86)	0.008
sPAP, mmHg	46 (14)	43 (14)	0.372
LVEF Simpson, %	52 (12)	51 (15)	0.510
RV free wall strain, %	20 (6.4)	22.3 (6.7)	0.292
RV free wall strain rate, 1/s	1.3 (0.4)	1.2 (0.3)	0.741
**Invasive hemodynamic measurements**			
sPAP, mmHg	43.5 (7.5)	44.7 (14.1)	0.619
dPAP, mmHg	16.3 (6.5)	18.6 (7)	0.157
mPAP, mmHg	26.8 (8.5)	28.5 (9.4)	0.402
mPCWP, mmHg	18.2 (6.9)	18.9 (7)	0.671
vRA, mmHg	15 (7)	19.5 (11.1)	0.071
mRA, mmHg	10.9 (5.2)	15 (8.1)	**0.022**
PVR, WU	2.6 (1.5)	3 (2.2)	0.301
DPG, mmHg	−2 (4.5)	−0.3 (4.9)	0.120
TPG, mmHg	8.6 (4.6)	9.6 (6.3)	0.393

*Values are numbers (%) or mean (standard deviation). Bold p-values are statistically significant*.

*NYHA, New York Heart Association; PCI, percutanous coronary intervention; CABG, coronary artery bypass graft; CIED, cardiac implantable eletronic device; eGFR, estimated glomerular filtration rate; NT-proBNP, N-terminales pro-B-type natriuretic peptide; EuroSCORE, European Sytem for Cardiac Operative Risk Evaluation; PH, pulmonary hypertension; TMVR, transcatheter mitral valve repair; TR, tricuspid regurgitation; EROA, effective regurgitant orifice area; RegVol, regurgitant volume; TV, tricuspid valve; TAPSE, tricuspid annulus plane systolic excursion; RV, right ventricle; FAC, fractional area change; RA, right atrium; sPAP, systolic pulmonary artery pressure; LVEF, left ventricular ejection fraction; dPAP, diastolic pulmonary artery pressure; mPAP, mean pulmonary artery pressure; mPCWP, mean pulmonary capillary Wedge pressure; vRA, v-wave pressure right atrium; mRA, mean pressure right atrium; PVR, pulmonary vascular resistance; WU, Wood units; DPG diastolic pulmonary pressure gradient; TPG, transpulmonary pressure gradient*.

### Invasive Hemodynamics and TR Reduction

Logistic regression analysis showed a significant relationship between mean RA pressure and ≥1 grade TR reduction in uni- and multivariate analysis (univariate: odds ratio 0.894, conf-interval 0.821–0.974, *p* = 0.010; multivariate: odds ratio 0.848, conf-interval 0.734–0.979, *p* = 0.025) and between PVR and ≥1 grade reduction in multivariate analysis (odds ratio 1.008, conf-interval 1.000–1.015, *p* = 0.047). sPAP, mPAP, and mPCWP and ≥1 grade reduction showed no significant relationship. No value showed a significant association with two or more grade reduction in uni- or multivariate logistic regression.

### TR Reduction and RV Remodeling

**Figure 2:**
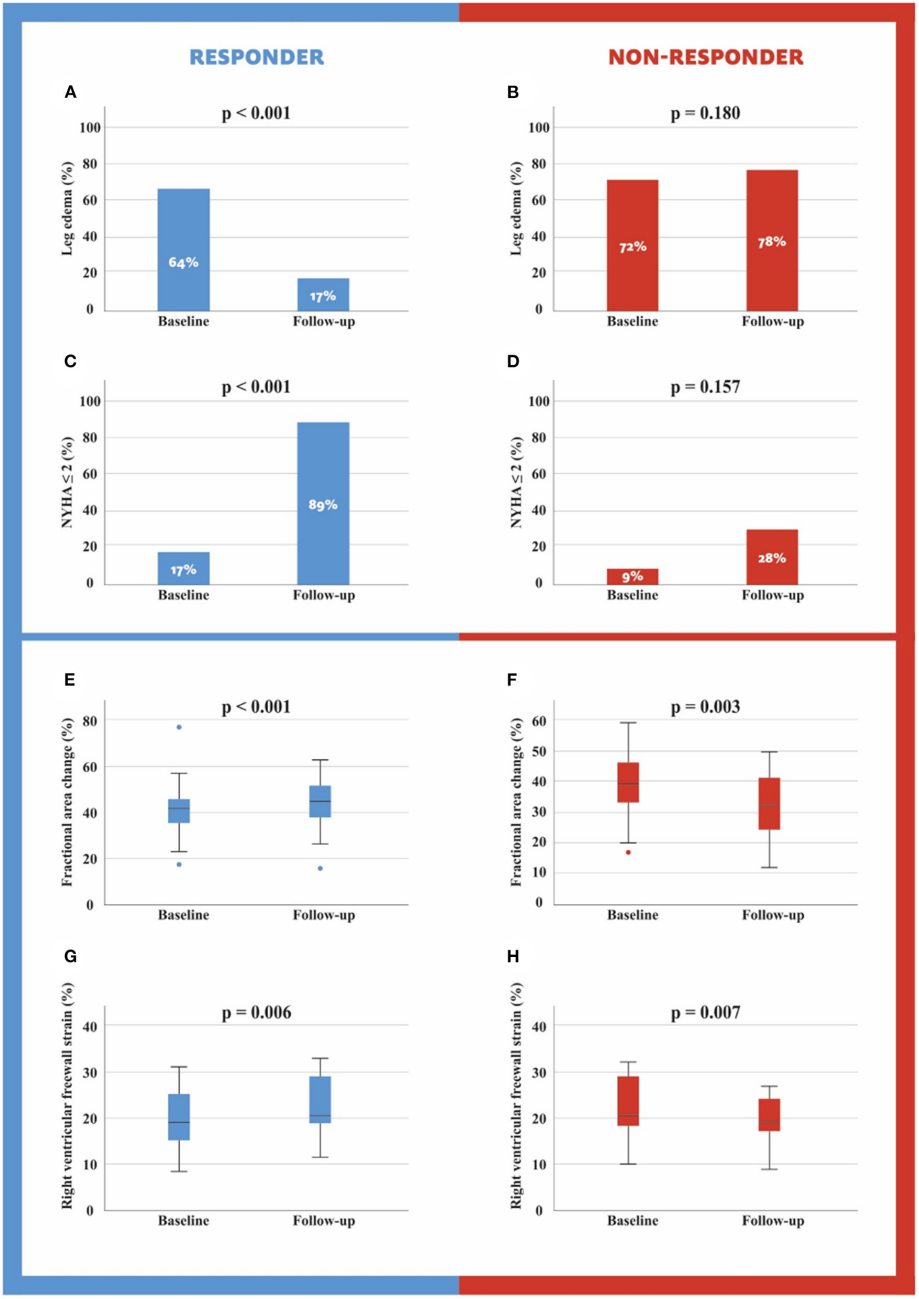
Leg edema and NYHA score at baseline and follow-up for responders and non-responders **(A–C)**. Fractional area change and right ventricular freewall strain at baseline and follow-up for responders and non-responders **(D–H)**.

Follow-up visits were performed at a mean of 229 days post TTVR for responders and 187 days post TTVR for non-responders. For patients undergoing reintervention, outcome data were obtained before reintervention. Leg edema and NYHA classification improved in the responder group significantly (64% to 17% for leg edema, *p* < 0.001 and 17% to 89% for NYHA ≤ 2, *p* < 0.001, Figures 2A,C) and did not change significantly in the non-responder group (72% to 78% for leg edema, *p* = 0.180 and 9% to 18% for NYHA ≤ 2, *p* = 0.157, Figures 2B,D). The following TR echocardiographic parameter were significantly reduced in the responder group: TR VC [16 (6) to 6 (3) mm, *p* < 0.001], TR EROA [0.75 (0.48) vs. 0.18 (0.14) cm^2^, *p* < 0.001] and TR RegVol [60 (27) vs. 15 (11) mL, *p* < 0.001] while in the non-responder group only TR RegVol decreased significantly [60 (28) vs. 48 (22) mL, *p* = 0.016] (Tables 3, 4).

**Table 3:** Logistic regression analysis for ≥1 and ≥2 grade TR reduction after TTVR and invasive hemodynamic parameters.

**≥1 grade TR reduction**						
	**Univariate**			**Multivariate**		
	**Odds ratio**	**95% Conf-interval**	***p***	**Odds ratio**	**95% Conf-interval**	***p***
sPAP	0.998	0.959–1.038	0.998	1.092	0.939–1.270	0.252
mPAP	0.977	0.918–1.039	0.458	0.898	0.681–1.184	0.445
mPCWP	0.936	0.866–1.012	0.098	0.984	0.827–1.171	0.856
mRA	0.894	0.821–0.974	**0.010**	0.848	0.734–0.979	**0.025**
PVR	1.005	0.999–1.012	0.090	1.008	1.000–1.015	**0.047**
**≥2 grade TR reduction**						
			**Univariate**			**Multivariate**		
	**Odds ratio**	**95% Conf-interval**	***p***	**Odds ratio**	**95% Conf-interval**	***p***
sPAP	1.009	0.978–1.041	0.561	1.036	0.945–1.136	0.454
mPAP	1.009	0.961–1.058	0.728	0.929	0.775–1.113	0.424
mPCWP	1.021	0.960–1.086	0.504	1.085	0.958–1.229	0.198
mRA	0.974	0.911–1.041	0.430	0.923	0.832–1.025	1.025
PVR	1.001	0.998–1.005	0.377	1.003	0.999–1.007	0.181

*sPAP, systolic pulmonary artery pressure; mPAP, mean pulmonary artery pressure; mPCWP, mean pulmonary capillary Wedge pressure; mRA, mean pressure right atrium; PVR, pulmonary vascular resistance. Bold p-values are statistically significant*.

**Table 4:** Comparison of baseline and follow-up data divided by responder and non-responder.

	**Responder**			**Non-responder**		
	**Baseline**	**Follow-up**	***p***	**Baseline**	**Follow-up**	***p***
NYHA ≤ 2	13 (17)	32 (89)	**<0.001**	3 (9)	5 (28)	0.157
Leg edema	48 (64)	6 (17)	**<0.001**	23 (72)	14 (78)	0.180
eGFR, mL/min	47 (22)	41 (16)	**0.006**	47 (16)	42 (20)	0.260
NT-proBNP, ng/L	4,200 (5,271)	2,540 (2,872)	**0.032**	2,231 (1,744)	3,660 (3,181)	0.096
RV basal diameter, mm	46.4 (6.2)	43.5 (7.5)	**0.001**	51.3 (11.1)	54.4 (8.6)	0.062
TV annulus, mm	40.2 (5.9)	38.3 (6.9)	**0.004**	45.6 (9.2)	46.2 (6.7)	0.690
TAPSE, mm	17 (5.3)	18.2 (4.7)	0.083	16.4 (5.3)	14.4 (5)	**0.001**
RV s', cm/s	10.8 (2.5)	11.7 (2.4)	**0.048**	9.1 (2)	9.1 (3)	0.927
FAC, %	38.6 (8.6)	44.3 (10)	**<0.001**	37.7 (9.3)	33.1 (9.8)	**0.003**
RA volume, ml	109 (42)	110 (49)	0.793	180 (78)	181 (83)	0.917
sPAP_echo_, mmHg	46 (13)	40 (10)	**0.003**	43.7 (14.2)	40 (8.6)	0.092
RV free wall strain, %	19.8 (6.6)	23.7 (5.6)	**0.006**	22.6 (6.7)	18.7 (4.5)	**0.007**
RV free wall strain rate, 1/s	1.2 (0.4)	1.4 (0.4)	**0.016**	1.2 (0.3)	1.1 (0.2)	0.281
RV free wall strain basal, %	18.2 (7.4)	24.2 (6.4)	**0.002**	20.7 (7.3)	19 (5.3)	0.393
RV free wall strain mid, %	20.5 (7.6)	25.1 (6.6)	**0.009**	22.7 (6.6)	19.4 (5.1)	**0.038**
RV free wall strain apical, %	20.6 (8.9)	21.7 (6.2)	0.550	24.5 (7.6)	17.8 (6.4)	**0.004**
TR grade ≥3	70 (93)	4 (11)	**<0.001**	31 (97)	16 (89)	0.564
TR Vena contracta, mm	16 (6)	6 (3)	**<0.001**	17 (4.8)	15 (4.8)	0.077
TR EROA, cm^2^	0.75 (0.48)	0.18 (0.14)	**<0.001**	0.85 (0.66)	0.69 (0.39)	0.158
TR RegVol, mL	60 (27)	15 (11)	**<0.001**	60 (28)	48 (22)	**0.016**
TV inflow gradient, mmHg	1.2 (0.6)	2.1 (1.1)	**<0.001**	1.4 (0.7)	2.8 (1.8)	**<0.001**

*Values are numbers (%) or mean (standard deviation). Bold p-values are statistically significant*.

*NYHA, New York Heart Association; eGFR, estimated glomerular filtration rate; NT-proBNP, N-terminales pro-B-type natriuretic peptide; RV, right ventricle; TV, tricuspid valve; TAPSE, tricuspid annulus plane systolic excursion; FAC, fractional area change; RA, right atrium; sPAP_echo_, systolic pulmonary artery pressure by echocardiography; TR, tricuspid regurgitation; EROA, effective regurgitant orifice area; RegVol, regurgitant volume*.

In the group of responders, RV basal diameter [46.4 (6.2) vs. 43.5 (7.5) mm, *p* = 0.001] and tricuspid valve (TV) annulus [40.2 (5.9) vs. 38.3 (6.9) mm, *p* = 0.004] decreased, while RV s' [10.8 (2.5) vs. 11.7 (2.4) m/s, *p* = 0.048], FAC [38.6 (8.6) vs. 44.3 (10) %, *p* < 0.001, Figures 2E,F], RV free wall strain [19.8 (6.6) vs. 23.7 (5.6) %, *p* = 0.006, Figures 2G,H] and RV free wall strain rate [1.2 (0.4) vs. 1.4 (0.4) 1/s, *p* = 0.016] increased significantly. Furthermore, RV free wall strain basal [18.2 (7.4) vs. 24.2 (6.4) %, *p* = 0.002] and RV free wall strain mid [20.5 (7.6) vs. 25.1 (6.6) %, *p* = 0.009] improved (Table 4).

And in the group of non-responders, TAPSE [16.4 (5.3) vs. 14.4 (5) mm, *p* = 0.001], FAC [37.7 (9.3) vs. 33.1 (9.8) %, *p* = 0.003] and RV free wall strain [22.6 (6.7) vs. 18.7 (4.5) %, *p* = 0.007] decreased significantly. Moreover, RV free wall strain mid [22.7 (6.6) vs. 19.4 (5.1) %, *p* = 0.038] and RV free wall strain apical [24.5 (7.6) vs. 17.8 (6.4) %, *p* = 0.004] deteriorated (Table 4).

RV functional parameters did not change significantly at follow-up when patients were divided into the different PH groups (Table 5).

**Table 5:** Comparison of right ventricular parameters at baseline and follow-up for different PH groups.

	**No PH**			**Postcapillary PH**			**Precapillary and combined PH**		
	**Baseline**	**Follow-up**	***p***	**Baseline**	**Follow-up**	***p***	**Baseline**	**Follow-up**	***p***
RV basal diameter, mm	47.4 (7.9)	47.7 (8.8)	0.773	48 (7.6)	47.7 (7.6)	0.754	45.4 (8)	43.9 (8.5)	0.592
TV annulus, mm	42.6 (7.1)	42.6 (8.7)	0.958	42 (7.8)	40.1 (5.6)	0.097	39.4 (6.3)	39.4 (6.8)	1.000
TAPSE, mm	18 (5)	17.3 (4.5)	0.471	17.3 (4.9)	17.8 (4)	0.463	16.3 (5.8)	17 (6.1)	0.592
RV s', cm/s	10.4 (2.2)	11.3 (2.9)	0.228	10.3 (2.1)	10.7 (2.4)	0.487	9.6 (3.1)	10.1 (2.9)	0.578
FAC, %	42.6 (6.7)	43.3 (10.4)	0.660	37.8 (8.8)	39.7 (10.9)	0.359	39.1 (8.5)	42.4 (11.4)	0.459
RA volume, ml	128 (59)	139 (58)	0.217	144 (77)	136 (65)	0.375	102 (42)	106 (93)	0.845
sPAP, mmHg	39.2 (9.7)	37.6 (6.9)	0.328	47.7 (13.1)	43.2 (12.1)	0.106	48.8 (15.3)	42 (8.2)	0.095
RV free wall strain, %	23.4 (5.3)	22.2 (4.9)	0.492	21.1 (5.7)	22.3 (5.7)	0.532	22.9 (9.2)	24.4 (6.2)	0.673
RV free wall strain rate, 1/s	1.4 (0.3)	1.3 (0.4)	0.292	1.3 (0.4)	1.3 (0.4)	0.503	1.2 (0.5)	1.4 (0.4)	0.323

*Values are numbers (%) or mean (standard deviation). Bold p-values are statistically significant*.

*PH, pulmonary hypertension; RV, right ventricle; TV, tricuspid valve; TAPSE, tricuspid annulus plane systolic excursion; FAC, fractional area change; RA, right atrium; sPAP_echo_, systolic pulmonary artery pressure by echocardiography; TR, tricuspid regurgitation; EROA, effective regurgitant orifice area; RegVol, regurgitant volume*.

### Clinical Endpoints and Outcome

**Figure 3:**
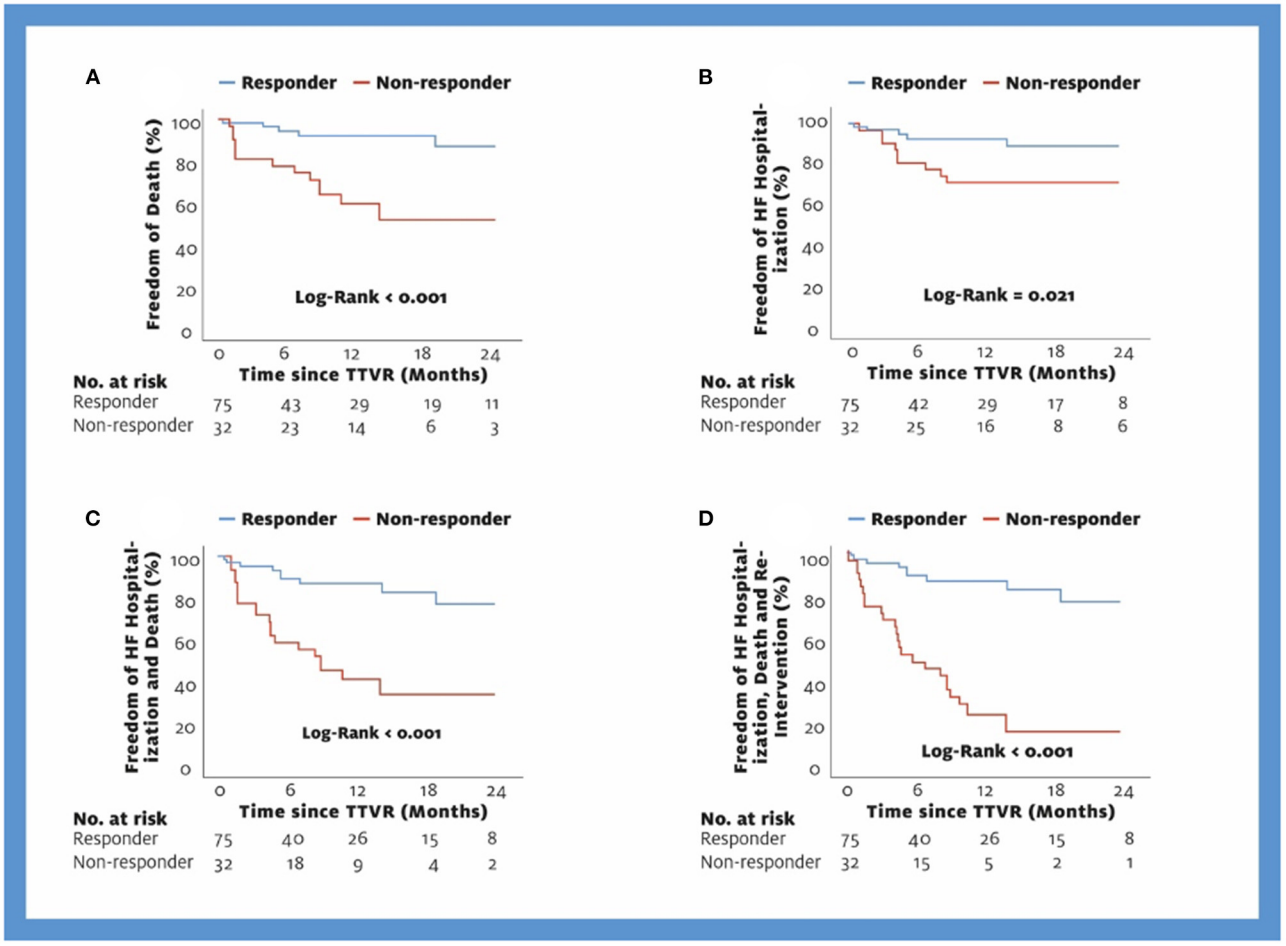
Kaplan-Meier Curves for the endpoints Death **(A)**, HF hospitalization **(B)**, Death and HF hospitalization **(C)**, and Death, HF hospitalization, Re-intervention **(D)**. TTVR, transcatheter tricuspid valve repair; HF, heart failure.

**Figure 4:**
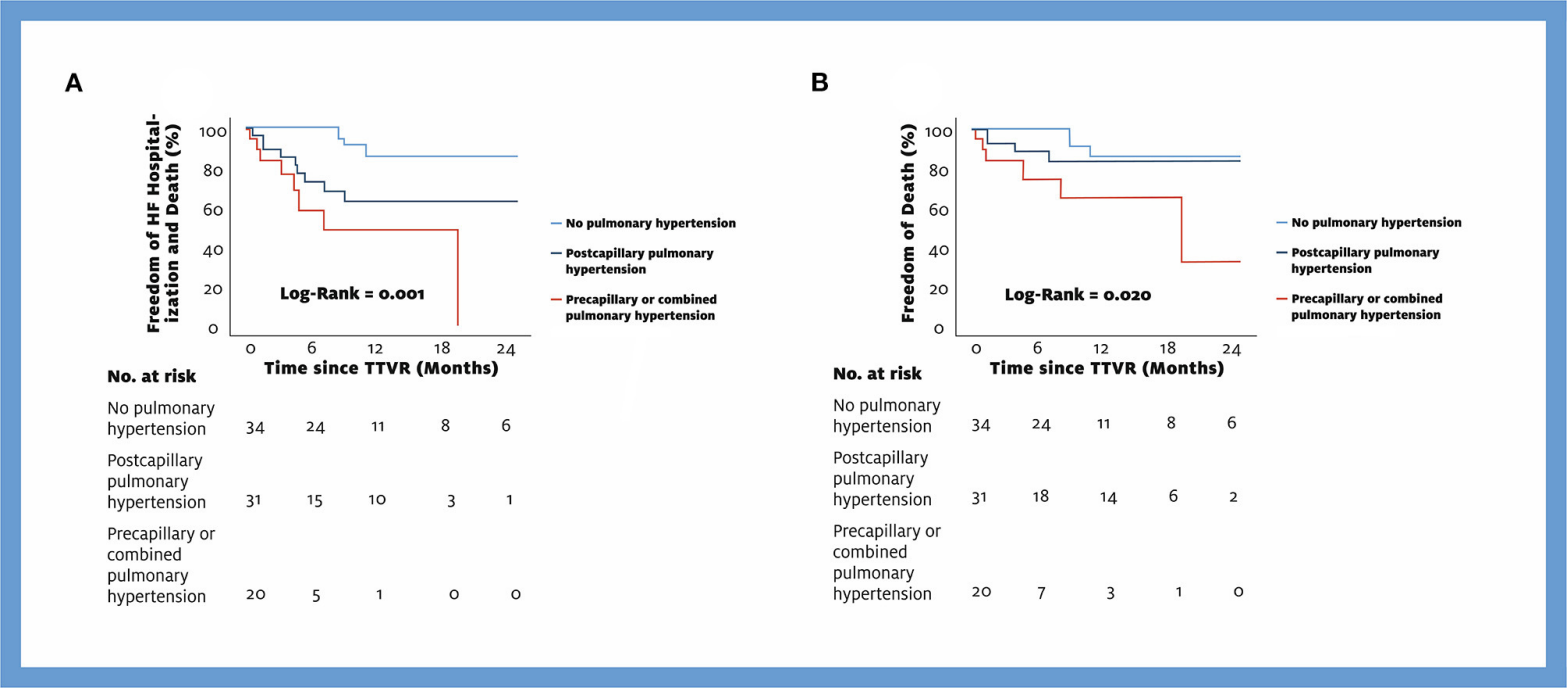
Kaplan-Meier Curves for the endpoints Death and HF hospitalization **(A)** and Death **(B)** by different PH groups. TTVR, transcatheter tricuspid valve repair; HF, heart failure.

A total of 39 events (18 deaths, 14 HF hospitalizations, 7 re-interventions) occurred during the observational period of 24 months [mean observational period 9 (8) months per patient]. In the responder group, 5 deaths, 5 HF hospitalizations, and no reintervention were recorded, whereas in the non-responder group, 13 patients died, 9 were hospitalized for HF, and 7 received reintervention. Rates for the combined endpoint of death, HF hospitalization, and re-intervention at 6 months, 1 year, and 2 years were for responders 11, 13, and 22%; and for non-responders, 51, 75, and 84% (log-rank: *p* < 0.001, Figure 3D). Similarly, a significant difference between responders and non-responders was found for the combined endpoint of death and HF hospitalization (22% vs. 66%, log-rank: *p* < 0.001, Figure 3C), for the isolated endpoint of death (12% vs. 47%, log-rank: *p* < 0.001, Figure 3A), and for the isolated endpoint of HF hospitalization (11% vs. 29%, log-rank: *p* = 0.021, Figure 3B). In addition, we analyzed outcome according to different PH groups. Rates for the combined endpoint of death and HF hospitalization at 6 months, 1 year, and 2 years for patients without PH were 0, 14, and 14%; for patients with postcapillary PH, 27, 37, and 37%; and for patients with precapillary or combined PH, 51, 51, and 100% (log-rank: *p* < 0.001, Figure 4A).

## Discussion

This prospective observational study divided TTVR patients into responders and non-responders according to pre-interventional hemodynamic assessment and procedural success. We were able to demonstrate three main findings: 1) Significant RV remodeling after TTVR, 2) Subsequent improvement or worsening of RV function depending on preinterventional hemodynamic status and procedural success, 3) Significantly lower mortality in patients with favorable hemodynamics and successful intervention, and 4) differences in outcome between the PH groups but no difference in RV remodeling.

### Patient Selection for TTVR

TR is a common disease with multiple causes that had long been treated only with guideline-directed medical therapy. The high prevalence of concomitant TR in various underlying diseases like left heart disease or PH makes patient selection a central issue for TTVR. Procedural success in TTVR is currently an ongoing matter of debate resulting in different definitions. Some authors advocate procedural success as a TR ≤ 2 after the procedure, whereas other authors define success based on the extent of reduction (12, 26). If procedural success is defined as TR ≤ 2 after TTVR, patients with massive or torrential TR have a lower procedural success rate and a higher HF hospitalization rate but a similar mortality rate compared with patients with severe TR (27). Our analysis demonstrated that reduction in TR was an important factor for a favorable outcome, regardless of baseline TR or residual TR after TTVR. We also analyzed invasive hemodynamic parameters and their predictive value for the success of the procedure (Table 3). Only right atrial mean pressure showed univariate and multivariate predictive value for TR reduction after TTVR. Elevated right atrial pressure could be a marker of advanced disease stage and should be considered in patient selection. Other values, such as mPAP or mPCWP, may not have prognostic significance because the number of patients in whom these values were strongly elevated was rather small. Furthermore, in our cohort, a substantial number of patients underwent concomitant TMVR (38%). TMVR is known to reduce pulmonary pressure and tricuspid regurgitation (28). In addition, patients with severe MR and TR who receive TMVR and TTVR might have a better outcome than patients who receive TMVR alone (29). Concomitant TMVR is a potential bias for our results, but responders and non-responders had no significant difference regarding the number of patients undergoing TMVR (responders: 35%, non-responders: 47%, *p* = 0.280).

Stocker et al. recently demonstrated that patients with precapillary PH who undergo TTVR have a worse outcome than patients without or with postcapillary PH (13). We also demonstrated that outcomes differed between PH groups and were worst in patients with combined or precapillary PH (Figure 4). Postcapillary PH due to left heart disease is a known factor for the occurrence of TR, but an additional precapillary PH component seems to worsen the outcome. Therefore, we included the PH group in our algorithm but also emphasized the success of the procedure. In our cohort, only two patients had precapillary PH and a TR reduction of one grade and were therefore assigned to the non-responder group. This can be explained by our screening for TTVR, which mostly excluded patients with precapillary PH due to early data of TTVR patients (30). The other 9 non-responders with a TR reduction of one TR grade met the criteria for combined PH according to guidelines (22). Still, pulmonary pressure and pulmonary resistance did not differ significantly between responders and non-responders (Table 6). This occurs because only patients with a decrease of one grade were placed in one of the groups according to PH, but still, the outcome in responders is much better. This suggests that, on the one hand, the benefit of a large TR reduction may overcome the poor prognosis of patients with PH. On the other hand, a TR reduction of one grade is not sufficient to compensate for the worse outcome of PH patients.

**Table 6:** Comparison of responder and non-responder data divided by baseline and follow-up examination.

	**Baseline**			**Follow-up**		
	**Responder**	**Non-responder**	***p***	**Responder**	**Non-responder**	***p***
NYHA ≤ 2	13 (17)	3 (9)	0.293	32 (89)	5 (28)	**<0.001**
Leg edema	48 (64)	23 (72)	0.432	6 (17)	14 (78)	**<0.001**
eGFR, mL/min	47 (19)	41 (16)	0.180	41 (20)	41 (16)	0.967
NT-proBNP, ng/L	3,785 (4,362)	4,083 (4,896)	0.796	2,370 (2,522)	3,932 (3,260)	0.099
RV basal diameter, mm	49 (8.3)	51.1 (10)	0.215	43.5 (7.5)	53.8 (8.8)	**<0.001**
TV annulus, mm	42.3 (7.2)	44.9 (8.6)	0.114	38.3 (6.9)	45.5 (7.5)	**<0.001**
TAPSE, mm	17.5 (5.5)	17 (5.7)	0.661	18.2 (4.7)	14.4 (5)	**0.003**
RV s', cm/s	10.6 (2.7)	9.3 (2.3)	**0.036**	11.6 (3)	8.9 (3)	**<0.001**
FAC, %	40.7 (9.1)	39 (10)	0.406	44.3 (10.1)	32.3 (10.4)	**<0.001**
RA volume, ml	122 (59)	171 (86)	**0.008**	110 (49)	174 (87)	**0.003**
sPAP, mmHg	46 (14)	43 (14)	0.372	39.6 (10.3)	39.6 (8.6)	0.971
RV free wall strain, %	20 (6.4)	22.3 (6.7)	0.292	22.8 (5.5)	19.1 (4.7)	**0.031**
RV free wall strain rate, 1/s	1.3 (0.4)	1.2 (0.3)	0.741	1.5 (0.5)	1.1 (0.2)	**0.002**
RV free wall strain basal, %	18.8 (7.4)	20.4 (7.2)	0.520	23.1 (6.7)	19.7 (5.7)	0.091
RV free wall strain mid, %	20.6 (7.4)	22.4 (6.5)	0.424	24.1 (6.3)	19.8 (5.2)	**0.025**
RV free wall strain apical, %	20.7 (8.4)	24 (7.6)	0.208	21.2 (6.0)	17.9 (6.6)	0.105
TR grade ≥3	70 (93)	31 (97)	0.468	4 (11)	16 (89)	**<0.001**
TR Vena contracta, mm	16 (5)	17 (5)	0.516	6 (3)	15 (5)	**<0.001**
TR EROA, cm^2^	0.77 (0.49)	0.85 (0.63)	0.769	0.18 (0.14)	0.68 (0.39)	**<0.001**
TR RegVol, mL	60 (26)	60 (27)	0.992	15 (11)	47 (22)	**<0.001**
TV inflow gradient, mmHg	1.2 (0.6)	1.4 (0.9)	0.354	2 (1.1)	2.8 (1.8)	**0.031**

*Values are numbers (%) or mean (standard deviation). Bold p-values are statistically significant*.

*NYHA, New York Heart Association; eGFR, estimated glomerular filtration rate; NT-proBNP, N-terminales pro-B-type natriuretic peptide; RV, right ventricle; TV, tricuspid valve; TAPSE, tricuspid annulus plane systolic excursion; FAC, fractional area change; RA, right atrium; sPAP_echo_, systolic pulmonary artery pressure by echocardiography; TR, tricuspid regurgitation; EROA, effective regurgitant orifice area; RegVol, regurgitant volume*.

Interestingly, despite the worse outcome of non-responders, both groups differ not much in terms of baseline characteristics. Non-responders had a significantly higher incidence of previous PCI, a larger RV, and RA. The EuroSCORE II was also higher in the non-responder group, but not significantly, whereas the recently introduced TRI-SCORE was able to show a significant difference (25). This is further suggestive that the EuroSCORE II may not be sufficiently prognostic for TR patients and may be inferior to the TRI-SCORE. Nevertheless, the small differences between responders and non-responders in baseline characteristics underline the impact of TR reduction and PH on the outcome.

### Right Ventricular Remodeling and Outcome

At echocardiographic follow-up, we observed a significant improvement in RV function and a decrease in RV size in the responder group (Table 4), similar to previous studies (11, 31, 32). However, for the first time, we also analyzed the group of non-responders who showed a decline in RV functional parameters (Table 4). This information supports the value of successful TTVR for TR patients. Interestingly, in the responder group, RV freewall strain increased more in the basal segments than in the apical segments. In contrast, it was reversed in the non-responder group concerning strain decrease (Table 4). The reason for this could be the indirect annuloplasty that occurs during TTVR. In the responder group, this annuloplasty combined with reduced volume overload after substantial TR reduction leads to reverse RV remodeling, especially in the large basal portions. In the group of non-responders, annuloplasty also takes place and probably has a protective effect on the basal parts of the RV, keeping them from deteriorating. However, due to volume overload following an incompletely repaired TR or an increased PVR, apical RV function deteriorates.

In addition to RV remodeling, we also analyzed the outcome with the endpoints of HF hospitalization, death, and re-intervention, also in combined analyses. We demonstrated a clear advantage for the responders (freedom of all endpoints after 2 years: responders 78%, non-responders: 16%, Figure 3D). Taramasso et al. compared TTVR patients with medical-treated patients in a propensity-matched analysis and demonstrated a survival rate of 64% in control patients and 77% in TTVR patients at 1 year (10). In our cohort, 60% of non-responders and 92% of responders survived after 1 year. The comparable outcome of our non-responders and the control patients by Taramasso et al. show that TR reduction of 1 grade in precapillary or combined PH is similar to no intervention in terms of survival. The higher survival of our responders compared with the TTVR group of Taramasso et al. can be explained by the assignment of procedural failures with no TR reduction to the non-responder group. Procedural failures also showed a significantly worse outcome in a separate analysis in the study by Taramasso et al. (10).

### Clinical Implications

We observed a significant clinical improvement in the responder group as measured by NYHA score, which demonstrated an increase in patients with NYHA ≤ II from 17 to 89% (Table 4, *p* < 0.001). In comparison, in the TRILUMINATE cohort, the number of NYHA ≤ II patients increased from 31 to 83% 1 year after TTVR (*p* < 0.0001) (11). Our responder patients seem to benefit even more compared to an entire TTVR cohort. However, in our non-responders, NYHA score did not change significantly (*p* = 0.157), consistent with the worse outcome of this group. We also observed no significant changes in the non-responder group in terms of leg edema (*p* = 0.180), while leg edema significantly improved in the responder group (*p* < 0.001). These clinical changes indicate that TTVR can help patients suffering from symptoms of right heart decompensation if PH is not precapillary or combined and at least 1 grade TR reduction is achieved. Finally, we can conclude that our study provides important insights into patient selection and TR reduction required for a good outcome. In addition, we were able to provide more detailed information on RV (reverse) remodeling after TTVR. Upcoming RCTs, such as the TRILUMINATE pivotal trial (unique identifier: NCT03904147), are eagerly awaited to clarify the impact of TTVR on TR patients.

## Limitations

There are several limitations to be considered in this study. We could not include all patients with TTVR from our center because invasive hemodynamic measurements were not available in all patients, mainly if TMVR was performed simultaneously. The changes in RV function and differences in outcome may also be attributable to concomitant TMVR, even though both groups had a similar repair rate (Table 1). Our procedural results are from a highly specialized center, nevertheless, patients from the beginning of TTVR were included. Therefore, the success rate of patients treated today might be higher. No echocardiography core laboratory was involved in image evaluation.

## Conclusion

TTVR patients divided into responders and non-responders by preinterventional hemodynamic assessment and procedural success show a marked difference in RV (reverse) remodeling and outcome. While RV function improves in responders, it deteriorates in non-responders. The endpoints of death, HF hospitalization, and reintervention were much more frequently reached by non-responders. Preprocedural hemodynamic assessment may help in patient selection. These encouraging results strengthen the usefulness of TTVR in routine clinical practice.

## Data Availability Statement

The raw data supporting the conclusions of this article will be made available by the authors, without undue reservation.

## Ethics Statement

The studies involving human participants were reviewed and approved by Ethik Kommision der Medizinischen Universität Wien. The patients/participants provided their written informed consent to participate in this study.

## Author Contributions

VD and GG: conception and design and analysis and interpretation of data. VD, GG, JM, and MS: drafting of the manuscript. GG, JM, MK, CD, KM, GH, KH, AB, MM, and GS: critical revision of the manuscript for important intellectual content. M-PW, PB, CH, GG, MA, AK, and CN: final approval of the submitted manuscript. All authors contributed to the article and approved the submitted version.

## Conflict of Interest

VD received consulting fees from Abbott, and educational grants from Edwards Lifesciences. JM received proctor fees from Abbott, consulting fees from Edwards Lifesciences, Boston Scientific, Shockwave Medical, and educational grants from Edwards Lifesciences. CH received proctor fees from Edwards Lifesciences and Boston Scientific, Educational grants from Abbott, Edwards Lifesciences, Boston Scientific, and Medtronic. MA received proctor/speaker/consulting fees from Abbott, Edwards, and Medtronic and institutional research funding (Edwards, Abbott, Medtronic, LSI). The remaining authors declare that the research was conducted in the absence of any commercial or financial relationships that could be construed as a potential conflict of interest.

## Publisher's Note

All claims expressed in this article are solely those of the authors and do not necessarily represent those of their affiliated organizations, or those of the publisher, the editors and the reviewers. Any product that may be evaluated in this article, or claim that may be made by its manufacturer, is not guaranteed or endorsed by the publisher.
